# Cytokines Inducing Bone Marrow SCA^+^ Cells Migration into Pancreatic Islet and Conversion into Insulin-Positive Cells *In Vivo*

**DOI:** 10.1371/journal.pone.0004504

**Published:** 2009-02-19

**Authors:** LuGuang Luo, John Z. Q. Luo, Fang Xiong, Mehrdad Abedi, Deborah Greer

**Affiliations:** 1 Center for Stem Cell Biology, Roger Williams Hospital, Providence, Rhode Island, United States of America; 2 PLME, Brown University, School of Medicine, Providence, Rhode Island, United States of America; University of Bremen, Germany

## Abstract

We hypothesize that specific bone marrow lineages and cytokine treatment may facilitate bone marrow migration into islets, leading to a conversion into insulin producing cells in vivo. In this study we focused on identifying which bone marrow subpopulations and cytokine treatments play a role in bone marrow supporting islet function in vivo by evaluating whether bone marrow is capable of migrating into islets as well as converting into insulin positive cells. We approached this aim by utilizing several bone marrow lineages and cytokine-treated bone marrow from green fluorescent protein (GFP) positive bone marrow donors. Sorted lineages of Mac-1^+^, Mac-1^−^, Sca^+^, Sca^−^, Sca^−^/Mac-1^+^ and Sca^+^/Mac-1^−^ from GFP positive mice were transplanted to irradiated C57BL6 GFP negative mice. Bone marrow from transgenic human ubiquitin C promoter GFP (uGFP, with strong signal) C57BL6 mice was transplanted into GFP negative C57BL6 recipients. After eight weeks, migration of GFP positive donor' bone marrow to the recipient's pancreatic islets was evaluated as the percentage of positive GFP islets/total islets. The results show that the most effective migration comes from the Sca^+^/Mac^−^ lineage and these cells, treated with cytokines for 48 hours, were found to have converted into insulin positive cells in pancreatic islets in vivo. This study suggests that bone marrow lineage positive cells and cytokine treatments are critical factors in determining whether bone marrow is able to migrate and form insulin producing cells in vivo. The mechanisms causing this facilitation as well as bone marrow converting to pancreatic beta cells still need to be investigated.

## Introduction

Studies have shown that bone marrow transplantation contributes to the recovery of islet β cell function through differentiation into islet β cells [1]. However, there are controversial arguments regarding a failure to document such transdifferentiation or a very low frequency of islet β cell differentiation [2]. Moreover, reports on bone marrow correcting experimental hyperglycemia in diabetic animals show that bone marrow initiates recipient β cell regeneration through donor derived endothelial cells in the pancreas rather than directly transdifferentiating into β cells [3], but others showed no improvement in hyperglycemia after bone marrow transplantation [4], [5]. Although this controversy has yet been resolved, bone marrow as a candidate for diabetes cell therapy has been considered and explored [6]–[8].

In this study, we proposed to identify whether specific bone marrow subpopulations and cytokine treatments play a critical role in bone marrow migration to pancreatic islets and conversion into islet cells in vivo. Specific populations of bone marrow can be induced into forming pancreatic islet β cells [9]–[11] and cytokines can be major regulators in influencing bone marrow conversion into pancreatic islet cells [12]. However, it is important to know which specific bone marrow population and cytokine treatment regulates bone marrow migration (homing) into islets after transplantation and whether migrated bone marrow can convert into islet cells. In this manuscript, we attempted to address these questions by testing GFP labeled bone marrow lineage cells in colocalized pancreatic islets with insulin positive cells under the following circumstances, a) GFP lineage cell populations from bone marrow cells only and, b) GFP lineage cells pre-cultured with cytokines, to evaluate whether these conditions facilitate bone marrow migration and conversion into pancreatic islet cells after transplantation.

## Materials and Methods

### Experimental Designs and Methods

#### Experimental Animals

C57BL/6-Tg (human ubiquitin C promoter-GFP) 30Scha/J mice (uGFP) and BL6-Tg(ACTB-EGFP)1Osb/J mice with an “enhanced” GFP (EGFP) cDNA, under the control of a chicken beta-actin promoter and cytomegalovirus enhancer (present in all tissues with the exception of erythrocytes and hair) emitting green under light excitation, were purchased from Jackson Laboratories (Bar Harbor, ME). Mice were certified to be pathogen free and housed in our animal facility with given ad libitum access to food and water. The eGFP mice were pancreatic donors and the uGFP mice were GFP labeled bone marrow donors. GFP negative C57BL mice were the recipients. Animal study protocols were approved by the Institutional Animal Care and Use Committee at Roger Williams Hospital.

#### Bone Marrow Transplantation

6–8 week old mice were used as donors or recipients. After sacrificing the mice and dissecting the femur, tibia and pelvic bones, bone marrow was obtained by flushing the bones using a syringe and a 22-gauge needle with PBS containing 5% heat-inactivated fetal calf serum (HI-FCS). After re-suspension in PBS without HI-FCS, cells were passed through a 40 µm cell strainer. Cell numbers were counted in crystal violet and viability was assessed by trypan blue staining. Whole bone marrow cells or selected populations of marrow cells, based on their surface markers, were injected intravenously by tail vein into each recipient. The dose of the cells infused was different in individual experiments and is mentioned in the results section. A photon producing linear accelerator (Elekta) was used for the radiation of recipient animals before each transplant. Radiation was given at a dose rate of 100 cGy per minute. The recipients received 500 cGy of whole body.

#### Pancreas Collection and Immunohistochemistry Staining

Specimens were collected after sacrificing the anesthetized mice by cervical dislocation. Excised pancreatic specimens were placed in freshly prepared PLP fixative solution (balanced phosphate solution with 2% paraformaldehyde, sodium m-periodate and L-lysine) for 2 hours at 4°C, with frequent agitation. Samples were then washed in a 7% sucrose buffer overnight followed by a 15% sucrose buffer wash for 2–3 hours and a 25% sucrose plus 10% glycerol buffer wash for another 2 hours, all at 4°C. They were then rinsed in PBS and embedded in tissue freezing medium (OCT), frozen and stored at −70°C until sectioning. Immunofluorescent staining was performed on 5 micron cryosections. For intracellular antigens, permeabilization was performed with 0.2% Triton X-100 for 20 minutes, followed by two PBS rinses. Sections were then blocked for 30 minutes with a 20% normal serum buffer. Sections were rinsed with PBS then incubated with anti-proinsulin, insulin and biotinylated anti-mouse CD45, rat monoclonal anti-CD34 (Abcam, Cambridge, MA) or Alexa Fluor 488 conjugated anti GFP antibodies (Molecular Probes, Eugene, OR) for 2 hours at room temperature, followed by 1 hour incubations with respective secondaries (Texas Red anti-guinea pig for insulin, Rhodamine anti-mouse for proinsulin and Alexa Flour streptavidin for CD45). GFP expression and antigens labeled by different fluorescence-conjugated antibodies were visualized by fluorescent microscopy (Axiovert w 135, Carl Zeiss, Oberko-chen, Germany). Additional staining with non-fluorescent DAB and Blue conjugated anti insulin, CD34 antibodies (Molecular Probes) in parallel sections were performed to confirm the fluorescent signals.

#### Cell Separation

Bone marrow was isolated from iliac bones, femur, and tibiae of GFP transgenic mice 6 to 8 weeks of age. Bone marrow cells were incubated with anti CD45-APC, anti c-Kit APC and anti-Sca-1-biotin SA- APC (Pharmingen, San Diego, CA). Cells positive and negative for individual markers were then sorted into different tubes with a high speed MoFlow cell sorter (Cytomation). For lineage negative separation, a low-density fraction (<1.077 g/cm2) was isolated on Nycoprep 1.077A (Accurate Chemical and Scientific Corporation). These cells were lineage depleted using magnetic beads from a Lineage Depletion kit (Miltenyi Biotec Inc. Auburn, CA). Cells were washed and counted after depletion. The population of sorted cells are displayed in Appendix S1.

#### Counting and Statistics

To count GFP positive islets in the pancreas, 12 sections, 5 microns thick and 250 microns apart, were made of each pancreatic specimen. The number of GFP positive islets and the total islets in each section were counted. The ratio between GFP positive islets and the total number of islets were determined as percentage of GFP positive islets in total islets. Data are presented as mean±one standard error of the mean. In the text, table, and figures, all data are presented as means±SEM. Most described experiments have been repeated three times. However, for figures and tables, only one experiment of the results is shown. Data used for graphical presentation and statistical analysis are expressed as per experiment. Data were analyzed by the ANOVA statistics program using a two factor analysis of variance of repeated measures. Post hoc comparisons among individual means were made by Tukey's t-test.

## Results

### GFPs are expressed in the pancreatic islets of uGFP transgenic mice and hematopoietic cells distribution in the pancreas

**Figure 1:**
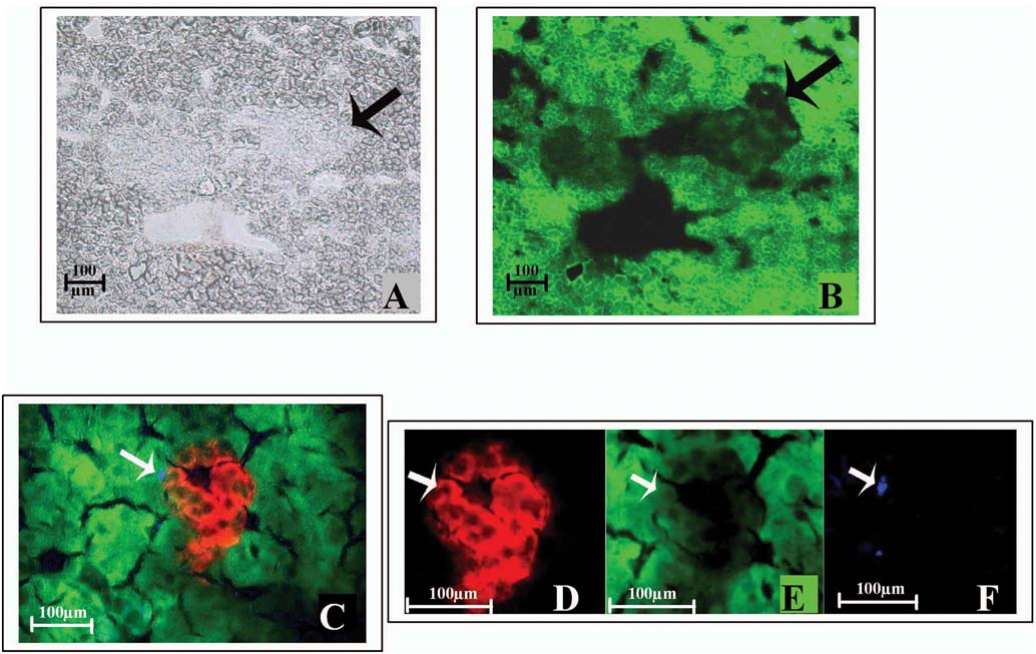
GFP signals in pancreatic islet of eGFP transgenic C57BL6 mice. GFP signals in pancreatic islets of eGFP transgenic positive mice were examined. A. Light microscopy image with the arrow indicating the tissue difference between a potential islet and the rest of the pancreatic tissue. B. The area indicated by the arrow in islet shows weak green fluorescence vs. surrounding area. C. Immunohistochemistry with insulin specific antibody indicated positive insulin cells (red) in islet but not yellow (mixture of green and red) and there is a CD45 cell (anti CD45 antibody blue) in the islet indicated by arrow. D. shows insulin positive cells only. E. shows weak GFP signals in islet areas vs. surrounding area. F. shows CD 45 positive cell only. A and B image magnification: ×5; C image magnification: ×20; D–F image magnification: ×40.

**Figure 2:**
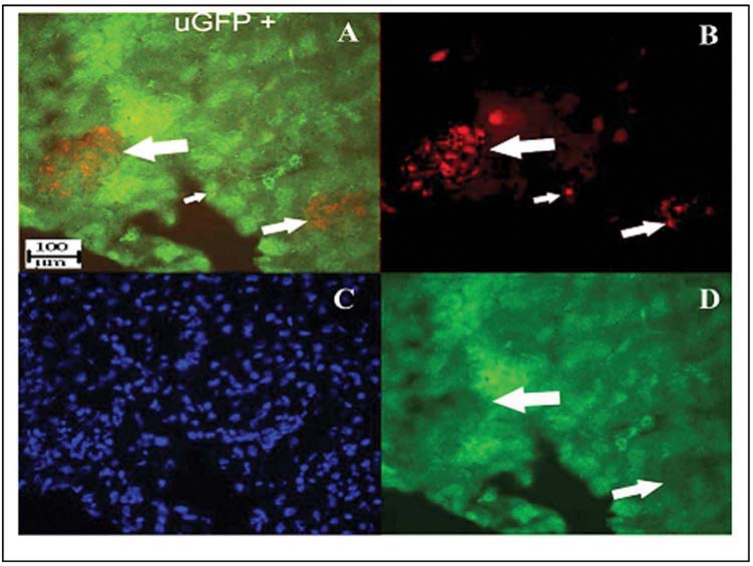
GFP expresses in pancreatic islet in uGFP transgenic C57BL6 mice. Clear GFP signals in pancreatic islets have been shown via immunohistochemistry of pancreatic sections from uGFP transgenic mice. A. Islet β cells have been identified by anti-insulin antibody (red, large arrow indicated) and the positive insulin islet mixture with green GFP fluorescence (small arrow indicated) combination color image; B. Islet β cells (red, arrows indicated) in red color image only; C. Nuclear staining with DAPI blue in blue color only; D. Positive GFP green in islet area (arrow indicated) in green color only, image magnification: ×40.

**Figure 3:**
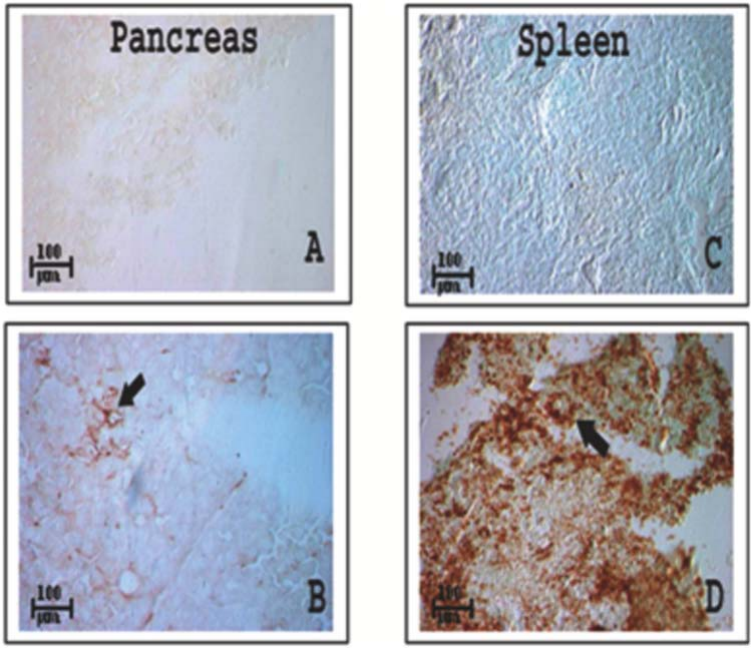
Distribution of CD45 cells in the pancreas and spleen of GFP negative mice. Identification of CD45+ cell migration into the pancreas and spleen was performed in GFP negative C57BL6 mice. A. Pancreatic tissue as negative control for immunohistochemistry with anti-CD45 specific antibody. B. CD45+ cells were found in pancreas (brown indicated by black arrow). CD45+ population in pancreas was about 4∼5/1000 pancreatic cells. C. Negative control of spleen. D. Spleen contains a large number of CD45+ cells 600∼700/1000 spleen cells while the amounts of bone marrow cells in pancreatic tissue (B) were low. Image magnification: ×5.

We detected weak signals of GFP in the pancreatic islet area of eGFP transgenic mice (Figure 1.). Un-homomorphy of GFP signals were found in (A) and (B) and these insulin positive areas were confirmed by fluorescent immunohistochemistry. There are significantly strong GFP positive expression in areas surrounding the insulin positive islet and CD45^+^ (C). The images further display individual channels for insulin (D), GFP (E) and CD45^+^ (F). GFP signals in uGFP transgenic mice are much stronger (Figure 2.) than GFP signals in pancreatic islet from eGFP transgenic mice (Figure 1). Since the pancreas is a more bone marrow resistant organ, there are low numbers of hematopoietic cells in the pancreas (about 4∼5/ 1000 pancreatic cells) as shown in Figure 3 (A) and (B), compared with an abundance of hematopoietic cells found in spleen (C) and (D). This suggests that bone marrow is more likely to home into the spleen than to the pancreas.

### Bone marrow cells migrate to pancreatic islets after transplantation

**Figure 4:**
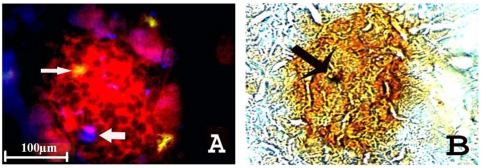
uGFP positive cell distribution in islet is CD 45 negative, but CD34 positive. A. Fluorescent immunohistochemistry with anti CD45 (blue) and insulin (red) antibodies were used to identify mice pancreatic GFP positive and CD45+ donor cells in GFP negative C57BL6 mice, which were transplanted with 25×10^6^ ml GFP positive WBM via tail vein after recipients had 500 cGy irradiation. GFP positive cells (indicated by narrow arrow) are CD45 negative. CD45 positive cell (indicated by large arrow) is GFP negative. B. CD34 positive cells were further confirmed by double labeling color immunohistochemistry with anti CD34 antibody (dark blue indicated by black arrow) in islet (brown) stained with anti-insulin antibody. Image magnification: ×40.

Utilization of uGFP transgenic mice as bone marrow donors allowed for tracking the labeled bone marrow migration into the recipient's pancreatic islets after transplantation (bone marrow 25×10^6^ cells) after 500 cGy (TBI). We found that infused bone marrow homes to the pancreatic islets, as indicated by anti insulin immunohistochemistry. These cells are also CD45 negative (Figure 4 A) and CD34 positive (B).

### Bone marrow subpopulation affects their migration into pancreatic islets

**Figure 5:**
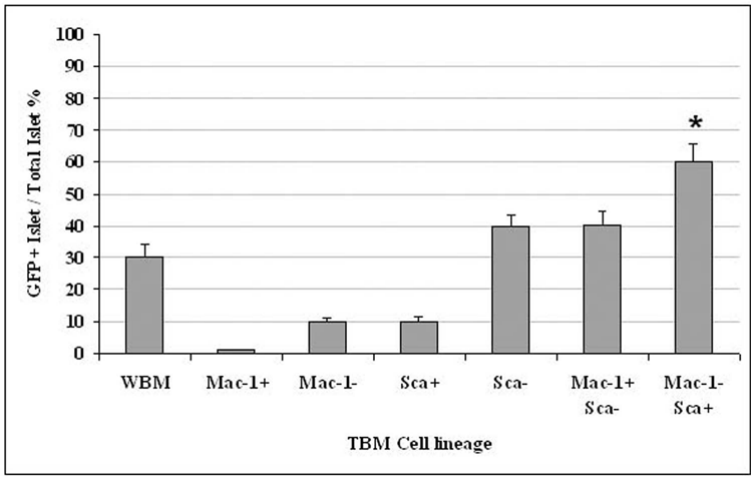
Lineage population affects bone marrow migration into pancreatic islets. Six different sorted GFP bone marrow populations were transplanted into GFP negative mice after 500 cGy TBI. The percentage of islets with positive GFP in total pancreatic islets was assayed. The result shows that favorable bone marrow population for migration into islet is Mac-1−/Sca+ vs. WBM (* = p<0.01). (TBM = tested bone marrow).

Lineage GFP positive bone marrow cells were isolated as described above in methods and 2.5×10^5^/ml cells were infused into recipients after 500 cGy irradiation. The positive GFP cells in pancreatic islets were analyzed 8 weeks after transplantation. There were six different cell lineages tested and whole bone marrow (WBM) was used as a control. The results show that the most favorable bone marrow population for migration to the islets is Mac-1−/Sca+ vs. WBM (Figure 5, * = p<0.01).

### Cytokines affect Sca+ lineage bone marrow differentiation into insulin positive cells in the recipient's islets

**Figure 6:**
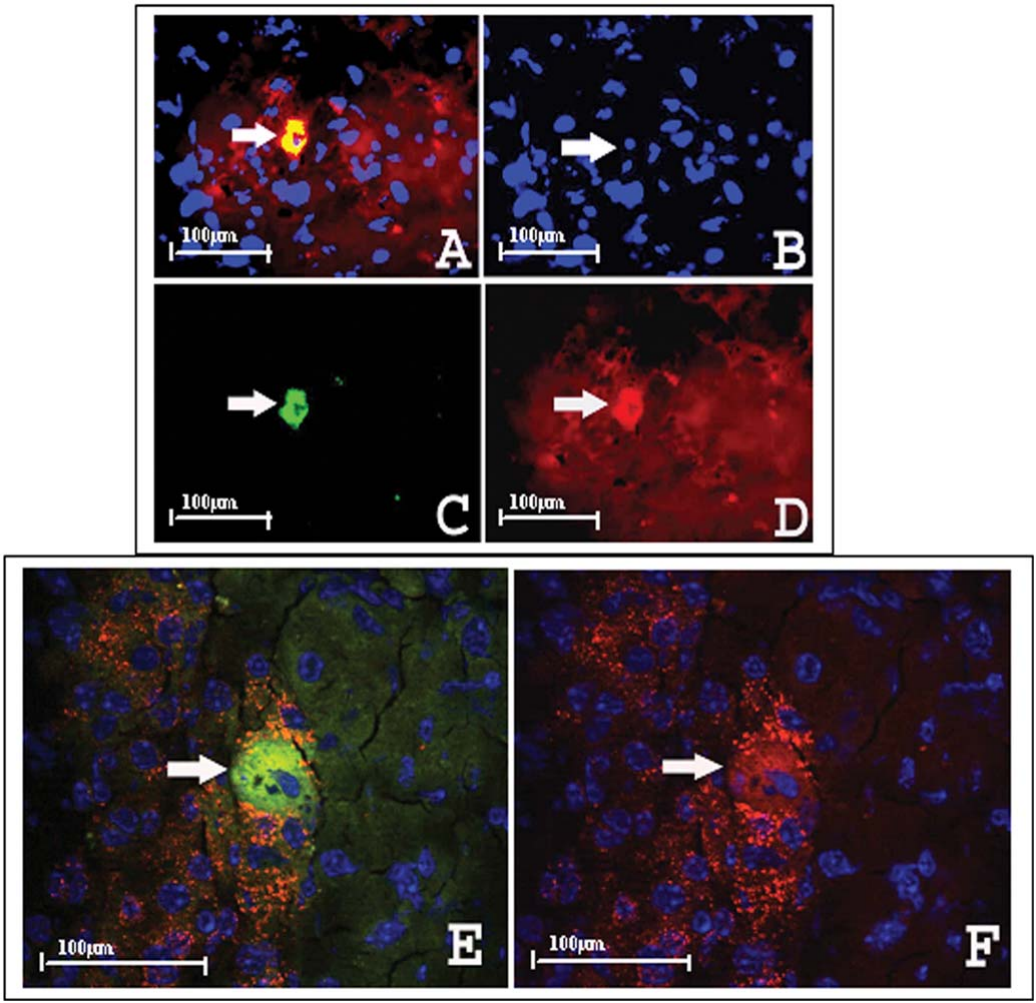
Cultured in presence of cytokines for 48 hours initiated BM differentiation to insulin positive cells in islet. A. Insulin positive cells were identified by insulin fluorescent immunohistochemistry as GFP positive (arrow indicated); B. Blue nucleus staining (DAPI) indicated by arrow; C. GFP positive cell indicated by arrow; D. The arrow indicates same cell with insulin positive staining. Image magnification: ×40; E. Anti-insulin antibody immunohistochemistry imaged under de-confocal microscopy to further identify differentiated cells. The arrow indicates the GFP positive cell is insulin positive with clear nucleus staining and F. insulin staining indicated by arrow and DAPI nuclear staining is blue. Image magnification: ×100.

Subpopulations of Sca+ cells were pre-cultured with cytokines IL-3 (50 U/ml), IL-6 (50 ng/ml), IL-11 (50 ng/ml) and steel (50 ng/ml) for 0, 24 and 48 hours. Cells positive for both GFP and insulin were only found in the recipient's pancreas from the 48 hour culture group as shown in Figure 5 immunohistochemistry images. Immunohistochemistry with specific anti-insulin antibody under de-confocal microscopy further confirmed that insulin positive cells were derived from positive GFP donor marrow. As viewed in Figure 6 A–D, cultures exposed to cytokines for 48 hours stimulated bone marrow Sca+ cells to differentiate into insulin positive cells in the pancreas. The amplified detail in the cytosol of insulin positive cells shows GFP staining colocalized with cytosol positive insulin granule staining in (E) and (F) (arrows indicate GFP positive and insulin granules in cytosol). The insulin positive granules can be clearly observed in the cellular cytosol of both GFP positive- insulin positive cells and insulin positive cells.

## Discussion

In this study, we found that GFP signals in eGFP animal pancreatic islets cells were particularly weak. This may be one reason that there were controversial reports for GFP in islets [3], [13], [14] . Therefore, in this study, we used uGFP animals as a bone marrow donor to avoid the weak GFP signal and focused on how bone marrow specific lineages, with the combination of cytokine treatment, influence bone marrow migration to islets and conversion into insulin positive cells in vivo.

We found that bone marrow cell migration into pancreatic tissue was restricted vs. migration into the spleen, as shown figure 3 (about 120 folds difference). More GFP positive cells were found in the outer perimeter of the islets than within the islet, it was 8.5∶1 or about 12% of total population, suggesting that bone marrow migration into islets is a critical step for bone marrow to contribute to islet β cell function and regeneration.

After testing six different sorted subpopulations of GFP positive bone marrow on GFP negative animals, we found that the bone marrow cell population Sca+/Mac-1− can migrate into islets more efficiently than WBM and the other five cell subpopulations. The mechanisms involved may rely on cellular surface chemokine receptor expression and pancreatic islet releasing factors, in which activation of chemokine receptors in bone marrow cells promotes cell migration into the islets [15]–[17]. On the other hand, Sca+ cell lineage has the potential to differentiate into insulin positive cells in vitro and in vivo [18]–[20]. Although we only found Sca+/Mac-1− favorable in this migration study, we are not excluding the possibility that other sub populations from bone marrow may also play a role in islet homing.

It has been reported that cytokine enriched bone marrow cultures alters bone marrow surface markers and improves bone marrow repopulation [21]. In this study, the Sca+/Mac-1 cell population was cultured with cytokines before transplantation for 0, 24 and 48 hours, revealing that Sca+ cells treated by cytokines for 48 hours induced differentiation into insulin positive cells in vivo. This suggests that cytokines are critical for bone marrow's participation in islet β cell function recovery and β cell regeneration. Cytokines tested in this study include IL-3, 6, 11 and steel factors in vitro. However, the levels of these factors, or others in vivo, could be different for individual responses to bone marrow transplantation, especially in damaged pancreatic animal models.

In summary, the current study provides evidence that transgenic mouse GFP gene control promoter, bone marrow subpopulation, and cytokines are critical factors to stimulate successful bone marrow migration and conversion into insulin-positive cells in islets in vivo. The phenomenon of cytokine treated bone marrow Sca+ subpopulations having the ability to migrate and potentially differentiate into insulin-positive islet cells still requires additional mechanistic studies to identify the factors behind this effect.

## Supporting Information

Appendix S1 (0.11 MB DOC)
