# Supplementary material for: Cytokines Inducing Bone Marrow SCA+ Cells Migration into Pancreatic Islet and Conversion into Insulin-Positive Cells In Vivo
Source: PLoS One. 2009 Feb 19;4(2):e4504. doi: 10.1371/journal.pone.0004504 (PMC2637986; doi:10.1371/journal.pone.0004504)
Supplement: Appendix S1 — (0.11 MB DOC) [file pone.0004504.s001.doc]

The graphs in this page represent that subpopulations sorted from whole bone marrow. Winders inside of graphs indicated positive cells which were sorted for transplantation. The cell population ranged from 10 ~ 20 percentage of total bone marrow cells.
